# Ischemic Stroke After Anticoagulation Discontinuation Following Apparent Resolution of Left Ventricular Thrombus: A Case Report

**DOI:** 10.7759/cureus.114727

**Published:** 2026-08-18

**Authors:** Shahad M Alhazmi, Abdulelah M Alhazmi, Omar S Badhawi

**Affiliations:** 1 Department of Internal Medicine, King Saud Medical City, Riyadh, SAU; 2 Department of Medicine and Surgery, Sulaiman Al Rajhi Colleges, Al Bukayriyah, SAU; 3 Department of Cardiology, King Saud Medical City, Riyadh, SAU

**Keywords:** apixaban, ischemic cardiomyopathy, ischemic stroke, left ventricular thrombus, transthoracic echocardiography

## Abstract

Left ventricular thrombus (LVT) is a serious complication of ischemic cardiomyopathy and anterior myocardial infarction, with an important risk of systemic embolism and stroke. Transthoracic echocardiography is commonly used for diagnosis and follow-up, but it may fail to detect residual thrombus in some high-risk patients. We report the case of a 64-year-old Saudi man with diabetes mellitus, hypertension, severe ischemic cardiomyopathy, and a known left ventricular apical thrombus after prior anterior myocardial infarction and multivessel coronary intervention. He was treated with apixaban and guideline-directed medical therapy. Follow-up echocardiography later showed no visible thrombus, and anticoagulation was discontinued. However, the patient subsequently developed an ischemic stroke with prolonged disability. This case highlights the diagnostic uncertainty that may remain after apparent resolution of LVT on echocardiography and emphasizes the need for careful clinical judgment before stopping anticoagulation in patients with severe left ventricular dysfunction and high embolic risk. Advanced imaging, such as cardiac magnetic resonance imaging or contrast-enhanced echocardiography, may be considered when diagnostic certainty is required.

## Introduction

Left ventricular thrombus (LVT) is a recognized and clinically significant complication following anterior myocardial infarction and in the setting of severe ischemic cardiomyopathy, with an associated risk of systemic embolization and ischemic stroke [1-4]. LVT formation is promoted by ventricular akinesia or dyskinesia, impaired left ventricular systolic function, and myocardial injury, which contribute to blood stasis and thrombus formation. Management generally includes anticoagulation with follow-up imaging to assess thrombus resolution [1,5].

Transthoracic echocardiography (TTE) is the most commonly used imaging modality for the detection and surveillance of LVT because of its widespread availability and noninvasive nature [5,6]. However, TTE has important limitations [5-8]. Its sensitivity is lower than that of cardiac magnetic resonance imaging (CMR), particularly for small, mural, or apical thrombi, and diagnostic uncertainty may remain in high-risk patients with severe left ventricular dysfunction and persistent apical akinesia [5-9]. Although CMR is more sensitive for detecting LVT, the role of advanced imaging before discontinuing anticoagulation should be individualized according to the patient’s clinical and thromboembolic risk.

In this case, we report a patient with a previously documented left ventricular apical thrombus in the setting of severe left ventricular systolic dysfunction and persistent apical akinesia who was treated with apixaban and guideline-directed medical therapy [1,6]. Follow-up contrast-enhanced TTE demonstrated apparent thrombus resolution, after which anticoagulation was discontinued. The patient subsequently developed an ischemic stroke. Although the clinical context raised concern for a cardioembolic mechanism related to recurrent or residual LVT, this mechanism could not be definitively established because CMR was not performed, and alternative mechanisms of ischemic stroke could not be completely excluded. This case highlights the diagnostic uncertainty that may remain after apparent LVT resolution on echocardiography and the potential value of more sensitive imaging in selected high-risk patients before decisions regarding discontinuation of anticoagulation.

## Case presentation

A 64-year-old Saudi man with diabetes mellitus and hypertension presented with acute anterior wall myocardial infarction (AWMI) in October 2021. Coronary angiography demonstrated triple-vessel disease. He declined coronary artery bypass grafting and underwent percutaneous coronary intervention (PCI) of the right coronary artery (RCA) with two drug-eluting stents and the left circumflex artery (LCX) with one drug-eluting stent in October 2021. The left anterior descending artery (LAD) chronic total occlusion and ramus intermedius lesion were managed medically. He later presented with non-ST-elevation myocardial infarction (NSTEMI) in August 2022 due to severe in-stent restenosis of the RCA and LCX and underwent intravascular ultrasound-guided PCI of the RCA in-stent restenosis with two additional drug-eluting stents. A planned reevaluation of the RCA stent and PCI of the LCX in-stent restenosis was not performed, and there was no plan for revascularization of the LAD chronic total occlusion.

**Figure 1:**
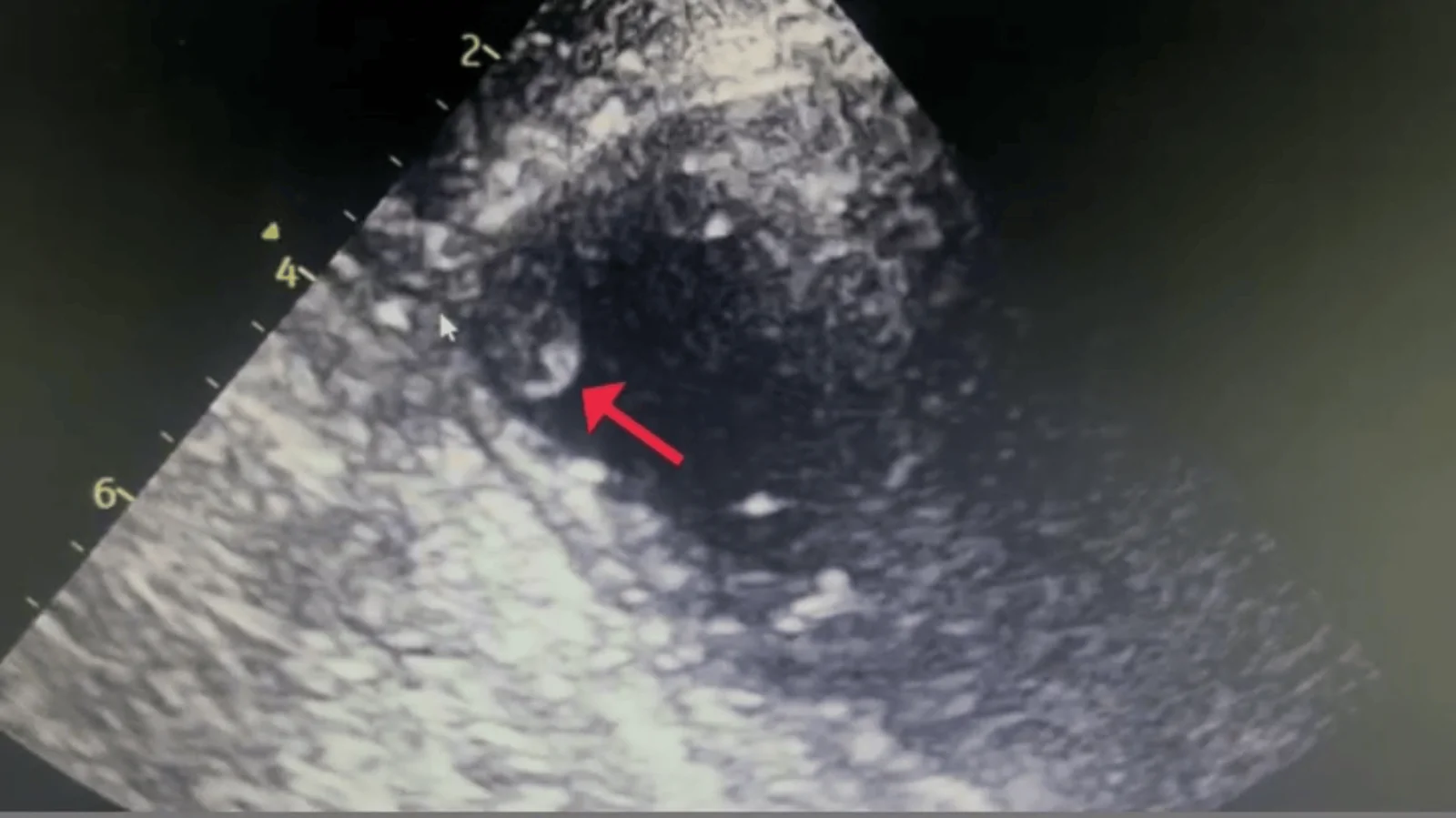
Transthoracic echocardiographic image demonstrating a left ventricular apical thrombus (red arrow) at the left ventricular apex in a patient with ischemic cardiomyopathy following anterior myocardial infarction.

**Figure 2:**
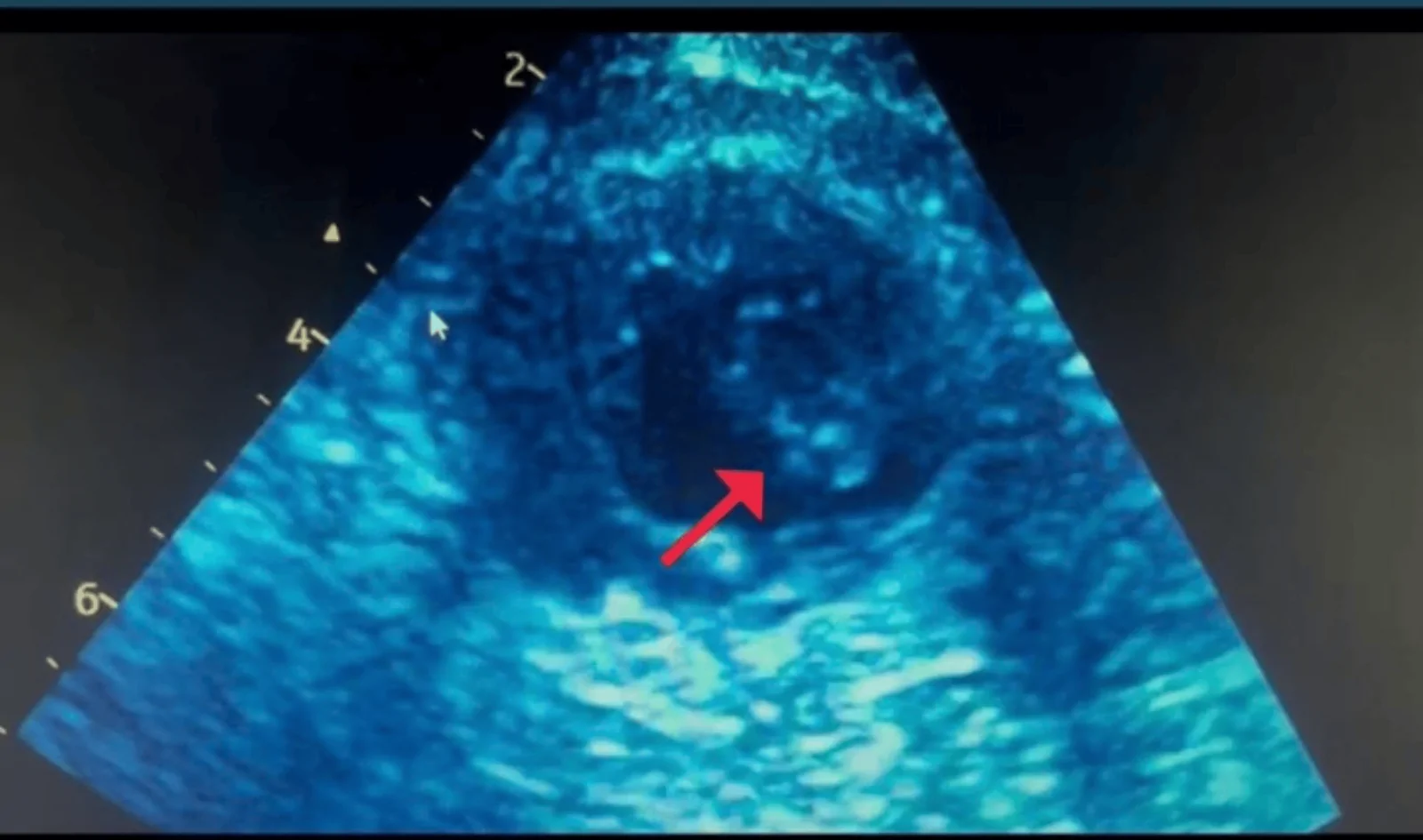
Additional transthoracic echocardiographic view demonstrating the left ventricular apical thrombus (red arrow), confirming the apical thrombus before anticoagulation.

Post-infarction, he developed ischemic cardiomyopathy with severely reduced left ventricular systolic function. Serial echocardiography demonstrated a left ventricular ejection fraction of approximately 30% and the presence of a left ventricular apical thrombus (Figures 1, 2).

He was treated with guideline-directed medical therapy for heart failure with reduced ejection fraction, including furosemide, sacubitril/valsartan, bisoprolol, spironolactone, empagliflozin, rosuvastatin, ezetimibe, and anticoagulation with apixaban 5 mg twice daily. His metabolic control was initially suboptimal, with a low-density lipoprotein cholesterol level of 5.3 mmol/L and an HbA1c level of 11.0% in June 2023. By January 2024, HbA1c improved to 8.1%.

On follow-up in May 2024, the patient was clinically stable, New York Heart Association class II, with a blood pressure of 140/65 mmHg and sinus rhythm. Anticoagulation with apixaban was continued. In October 2024, echocardiography confirmed heart failure with reduced ejection fraction (left ventricular ejection fraction (LVEF): 25%) with a persistent LVT.

**Figure 3:**
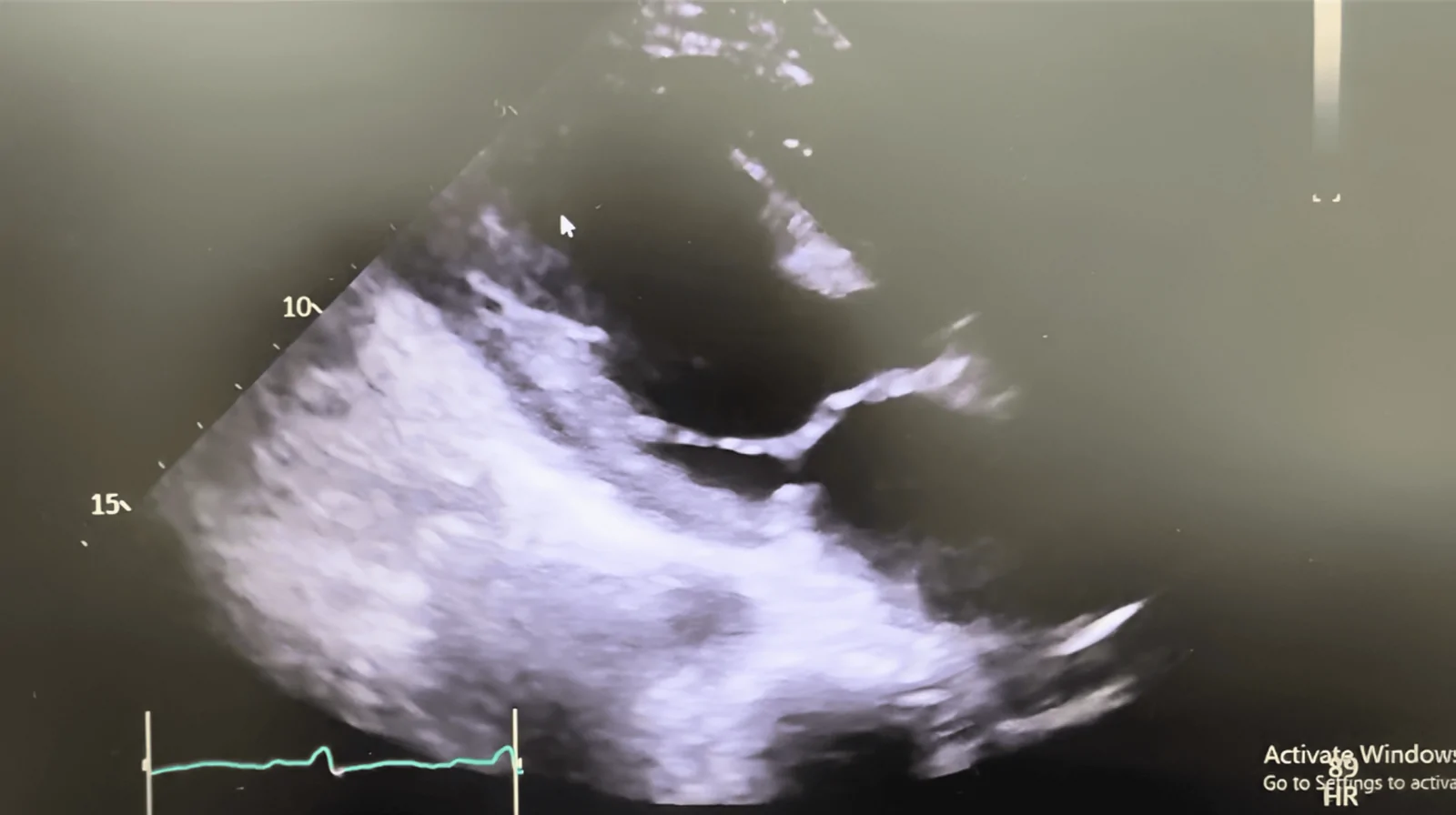
Follow-up transthoracic echocardiographic image obtained in August 2025 demonstrating severe left ventricular systolic dysfunction without a visible residual left ventricular apical thrombus. This image represents the non-contrast follow-up view; the clinical echocardiography report also documented no residual thrombus on contrast-enhanced imaging.

In August 2025, follow-up TTE demonstrated a moderately dilated left ventricle with persistent severe systolic dysfunction (LVEF 25-30%), septal akinesia, myocardial thinning, and scarring. No residual LVT was identified on TTE, including contrast-enhanced imaging. A representative follow-up echocardiographic image is shown in Figure 3. In view of the apparent resolution of the LVT, apixaban was discontinued. The patient remained clinically stable without significant cardiac symptoms.

**Figure 4:**
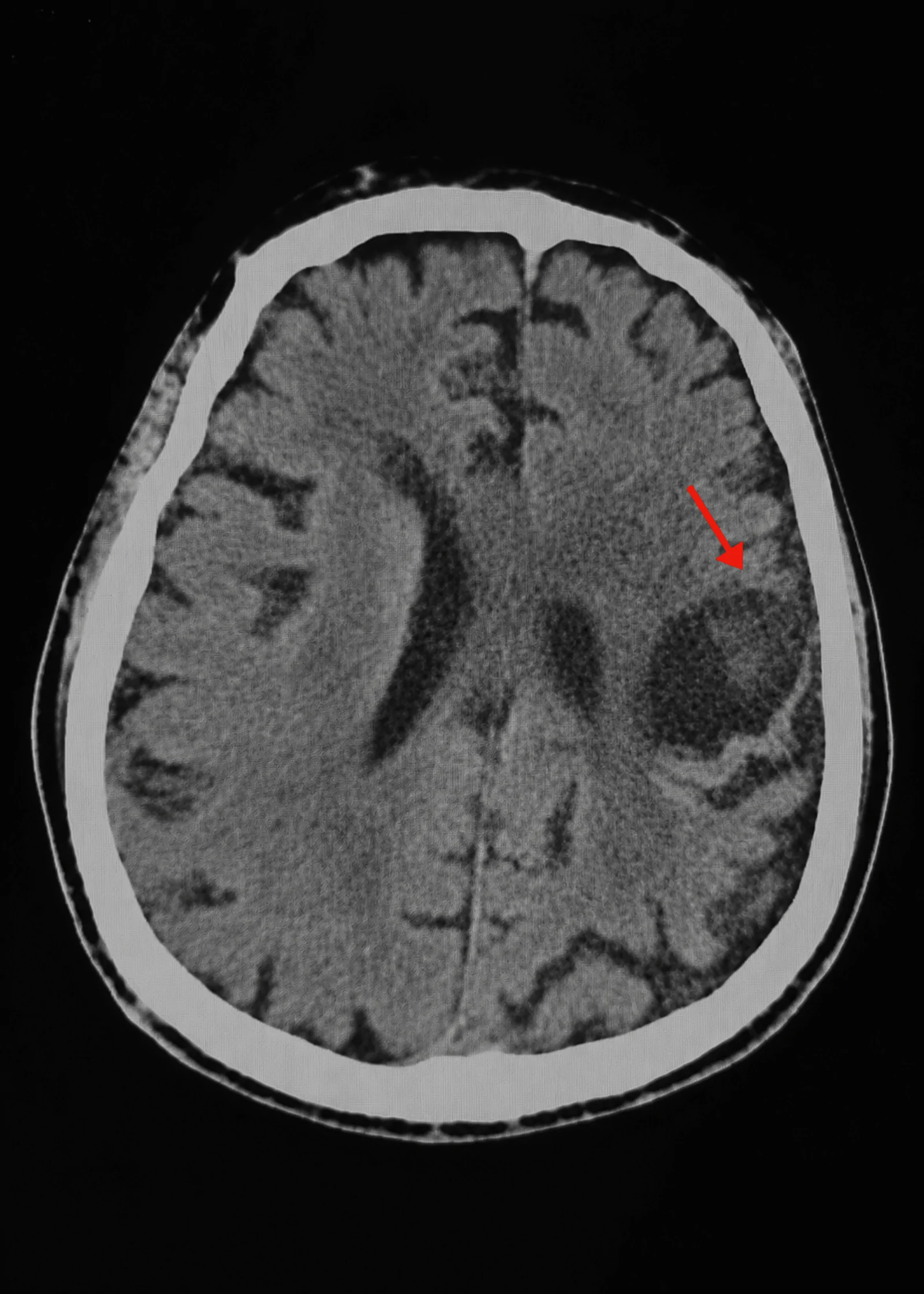
Non-contrast CT of the brain demonstrating a left frontoparietal chronic infarction involving the left middle cerebral artery territory, indicated by the red arrow.

Subsequently, the patient experienced an ischemic stroke resulting in major neurologic disability and prolonged hospitalization. This event occurred after anticoagulation had been discontinued following the apparent echocardiographic resolution of the LVT. Non-contrast CT of the brain demonstrated a left frontoparietal chronic infarction involving the left middle cerebral artery territory, as shown in Figure 4.

This case illustrates the diagnostic and therapeutic challenges of LVT in a patient with severe ischemic cardiomyopathy. Although follow-up TTE demonstrated apparent thrombus resolution, including no residual thrombus reported on contrast-enhanced imaging, the patient subsequently experienced a disabling ischemic stroke after anticoagulation was discontinued. This clinical course highlights the diagnostic uncertainty that may remain after apparent LVT resolution on TTE in selected high-risk patients and underscores the importance of individualized reassessment before discontinuation of anticoagulation.

## Discussion

This case underscores a critical diagnostic challenge in patients with severe ischemic cardiomyopathy and prior LVT: whether a negative transthoracic echocardiogram is sufficient to exclude persistent thrombus and safely discontinue anticoagulation [1,3,5,6]. TTE is the most widely used imaging modality for the detection and surveillance of LVT due to its accessibility, noninvasive nature, and real-time assessment of ventricular function [5,6]. However, its sensitivity is consistently lower than that of CMR, particularly for small, mural, or apical thrombi, and false-negative results can occur in high-risk patients with severe left ventricular dysfunction, akinetic segments, and extensive myocardial scar [5-9].

Recent systematic reviews and meta-analyses have demonstrated the limited sensitivity of TTE for detecting LVT compared with CMR [8,9]. Contrast-enhanced CMR has demonstrated substantially greater diagnostic sensitivity than TTE, including in studies using surgical or pathological validation [10]. These findings support the use of CMR when diagnostic uncertainty persists, particularly in high-risk patients in whom confirmation of thrombus resolution may influence decisions regarding anticoagulation [1,6,8,10].

The patient in this case had multiple high-risk features for LVT formation and recurrence, including prior AWMI, a severely reduced ejection fraction of 25-30%, apical akinesis, and extensive septal scarring [1-6]. The initial echocardiogram clearly identified a left ventricular apical thrombus, and anticoagulation with apixaban was initiated, consistent with contemporary management recommendations [1,6].

Follow-up TTE in August 2025 reported no visible thrombus, and anticoagulation was discontinued [1,6]. However, the patient subsequently developed an ischemic stroke with major disability. Although the prior LVT, persistent apical akinesis, and severe left ventricular systolic dysfunction raised concern for a possible cardioembolic source, persistent or recurrent thrombus was not confirmed, and alternative stroke mechanisms could not be excluded [1-3,5-9].

Apixaban and other direct oral anticoagulants have emerged as potential alternatives to warfarin for the treatment of LVT [1,3,6]. Several reports have described successful LVT resolution with apixaban [4,5]. However, the evidence base for direct oral anticoagulants remains less established than that for warfarin [1,6]. In this patient, the clinical course raises the possibility of persistent or recurrent LVT despite apparent resolution on TTE; however, this was not confirmed, and a definitive causal relationship with the subsequent ischemic stroke cannot be established [5-9].

The timeline in this case illustrates a critical learning point: apparent thrombus resolution on TTE may not completely eliminate diagnostic uncertainty regarding residual or recurrent thrombus in high-risk patients [1,6,8,10]. The decision to discontinue anticoagulation followed apparent thrombus resolution on TTE, including contrast-enhanced imaging, without confirmation by CMR [1,6,8,10]. In patients with previously documented LVT, severe left ventricular dysfunction, and residual apical dysfunction, more sensitive imaging, such as CMR, may be considered when diagnostic uncertainty persists before discontinuation of anticoagulation [1,6,8,10].

From a practical perspective, this case highlights the importance of maintaining a cautious approach when managing anticoagulation in patients with high embolic risk [1,6]. Clinicians should weigh the risk of recurrent thrombus formation and embolism against the risk of bleeding and consider advanced imaging for further assessment when uncertainty regarding thrombus resolution persists before discontinuing anticoagulation [1,6,8,10]. In patients for whom CMR is not feasible, contrast-enhanced echocardiography may be a useful alternative, although its sensitivity remains lower than that of CMR [6,8,10].

## Conclusions

LVT remains an important complication of ischemic cardiomyopathy and is associated with a significant risk of systemic embolization and ischemic stroke. Although TTE is widely used for diagnosis and follow-up, its limited sensitivity, particularly for small, mural, or apical thrombi, may leave residual diagnostic uncertainty despite an apparently negative study.

This case highlights the importance of individualized reassessment of thromboembolic risk before discontinuing anticoagulation, even when apparent thrombus resolution is reported on echocardiography. Persistent severe left ventricular systolic dysfunction and regional wall-motion abnormalities may continue to provide a substrate for thrombus formation. Advanced imaging, particularly CMR, may be considered when diagnostic uncertainty persists despite negative echocardiographic findings, especially in patients with persistent severe left ventricular dysfunction and apical akinesia. Decisions regarding the duration or discontinuation of anticoagulation should be individualized, balancing thromboembolic and bleeding risks and the underlying ventricular substrate.
